# Vectors Based on Modified Vaccinia Ankara Expressing Influenza H5N1 Hemagglutinin Induce Substantial Cross-Clade Protective Immunity

**DOI:** 10.1371/journal.pone.0016247

**Published:** 2011-01-24

**Authors:** Annett Hessel, Michael Schwendinger, Georg W. Holzer, Klaus K. Orlinger, Sogue Coulibaly, Helga Savidis-Dacho, Marie-Luise Zips, Brian A. Crowe, Thomas R. Kreil, Hartmut J. Ehrlich, P. Noel Barrett, Falko G. Falkner

**Affiliations:** 1 Department of Virology, Baxter Bioscience, Biomedical Research Center, Orth/Donau, Austria; 2 Department of Immunology, Baxter Bioscience, Biomedical Research Center, Orth/Donau, Austria; 3 Department of Animal Models, Baxter Bioscience, Biomedical Research Center, Orth/Donau, Austria; 4 Department of Bacteriology, Baxter Bioscience, Biomedical Research Center, Orth/Donau, Austria; 5 Global R&D Vaccines, Baxter Bioscience, Biomedical Research Center, Orth/Donau, Austria; McMaster University, Canada

## Abstract

**Background:**

New highly pathogenic H5N1 influenza viruses are continuing to evolve with a potential threat for an influenza pandemic. So far, the H5N1 influenza viruses have not widely circulated in humans and therefore constitute a high risk for the non immune population. The aim of this study was to evaluate the cross-protective potential of the hemagglutinins of five H5N1 strains of divergent clades using a live attenuated modified vaccinia Ankara (MVA) vector vaccine.

**Methodology/Principal Findings:**

The replication-deficient MVA virus was used to express influenza hemagglutinin (HA) proteins. Specifically, recombinant MVA viruses expressing the HA genes of the clade 1 virus A/Vietnam/1203/2004 (VN/1203), the clade 2.1.3 virus A/Indonesia/5/2005 (IN5/05), the clade 2.2 viruses A/turkey/Turkey/1/2005 (TT01/05) and A/chicken/Egypt/3/2006 (CE/06), and the clade 2.3.4 virus A/Anhui/1/2005 (AH1/05) were constructed. These experimental live vaccines were assessed in a lethal mouse model. Mice vaccinated with the VN/1203 hemagglutinin-expressing MVA induced excellent protection against all the above mentioned clades. Also mice vaccinated with the IN5/05 HA expressing MVA induced substantial protection against homologous and heterologous AH1/05 challenge. After vaccination with the CE/06 HA expressing MVA, mice were fully protected against clade 2.2 challenge and partially protected against challenge of other clades. Mice vaccinated with AH1/05 HA expressing MVA vectors were only partially protected against homologous and heterologous challenge. The live vaccines induced substantial amounts of neutralizing antibodies, mainly directed against the homologous challenge virus, and high levels of HA-specific IFN-γ secreting CD4 and CD8 T-cells against epitopes conserved among the H5 clades and subclades.

**Conclusions/Significance:**

The highest level of cross-protection was induced by the HA derived from the VN/1203 strain, suggesting that pandemic H5 vaccines utilizing MVA vector technology, should be based on the VN/1203 hemagglutinin. Furthermore, the recombinant MVA-HA-VN, as characterized in the present study, would be a promising candidate for such a vaccine.

## Introduction

Influenza A viruses infect, among other hosts, aquatic birds, poultry, swine and humans [1]. Whereas in aquatic birds the infection is asymptomatic, in humans influenza infection can cause severe symptoms. The highly pathogenic avian influenza (HPAI) viruses are considered candidates for a new pandemic. H5N1 is one of the most pathogenic subtypes and has caused more than 500 symptomatic infections worldwide, of which more than 300 were lethal [2]. So far, the H5N1 influenza subtype has not circulated in the human population. If H5N1 influenza viruses become transmittable from human-to-human a new pandemic is likely to occur. Therefore, the development of safe and effective vaccines has high priority. Since the exact subtype and clade of a potential future pandemic strain is not known, broad cross-protection is a highly desirable feature of any pre-pandemic vaccine. The key to successful vaccine design is understanding the cross-reactivity between the genetically distinct H5N1 strains.

As described in previous studies, inactivated vaccines containing the HA of clade 1 and 2.1 H5N1 influenza viruses display significant cross protective potential [3]–[5]. Cross-clade protection was also shown previously using virus like particles (VLPs) containing the HA, NA and M1 proteins of IN5/05 and VN/1203 [5], [6]. Furthermore, non-replicating vaccinia vectors including MVA may be a good alternative for cross reactive pandemic influenza vaccines. MVA is a highly attenuated strain of vaccinia virus with a long-standing safety record [7], [8] expressing foreign genes efficiently and inducing effective immune responses [9], [10]. In previous studies, a clade 1 MVA-H5 vaccine could protect mice against challenge with a clade 2.1 virus [11], [12] and the same vector conferred protection against homologous and heterologous H5N1 influenza virus infections also in macaques [13], [14]. Furthermore, a candidate clade 1 H5N1 vaccine based on defective vaccinia induced complete protection from lethal homologous virus challenge and also full cross-protection against clade 0 and 2 challenge viruses [15] and a pandemic H1N1 live vaccine based on MVA was highly immunogenic and protected mice in active and passive immunizations [16].

This study extends previous findings by investigating also the cross-protective potential of the HAs of more distantly related H5 viruses including clades 2.2 and 2.3.4 represented by the strains A/turkey/Turkey/01/05, A/Chicken/Egypt/3/06 (clade 2.2.) and A/Anhui/1/2005 (clade 2.3.4). This is particularly important, because the current focus of H5N1 activity is Egypt. In 2009 and 2010 more than 50% of cases worldwide have occurred in this country. To allow for direct comparison, the different HA genes were expressed by MVA vectors that were used as experimental live vaccines against H5N1 challenge. Protection of mice and induction of antibodies were analyzed after single dose vaccinations. Additionally, the role of T cell responses in cross-protection was analyzed.

## Materials and Methods

### Ethics statement

All animal experiments were reviewed by the Baxter Bioscience Institutional Animal Care and Use Committee (IACUC Vienna/Orth) and approved by internal animal welfare officers (Experiment ID 08/06/NÖ). Animal experiments were conducted in accordance with Austrian laws on animal experimentation and approved by Austrian regulatory authorities (permit number the Government of Lower Austria, LF1-TVG-25/010-2006). Experiments were conducted according to guidelines set out by the Association for Assessment and Accreditation of Laboratory Animal Care International (AAALAC). Animals were housed according to EU guidelines, in housing facilities accredited by the AAALAC.

### Cells and viruses

DF-1 (CRL-12203) cells were obtained from the American Type Culture Collection (ATCC). Cells were cultivated in DMEM (Biochrom AG) containing 5% fetal calf serum (FCS). Chicken embryo cells (CEC) were produced in house and cultivated in Med199 (Gibco, Inc.) containing 5% fetal calf serum (FCS). The influenza viruses A/Vietnam/1203/2004 (H5N1, CDC #2004706280) and A/Indonesia/5/05 (H5N1, CDC #2005740199) were kindly provided by the Centers for Disease Control and Prevention (CDC, Atlanta, USA). The A/turkey/Turkey/1/05(H5N1) virus was kindly provided by National Institute for Medical Research (NIMR). The influenza A/Anhui/5/2005 strain was generated by reverse genetics procedures and was provided by Green Hills Biotechnologies, Vienna. The vaccinia virus strain Lister/Elstree (VR-862) was obtained from the ATCC. The basis of the Lister constructs was the subcloned virus vpDW-862/Elstree. The MVA strain (MVA 1974/NIH clone 1) was kindly provided by B. Moss (National Institutes of Health).

### Cloning of the hemagglutinin genes

*pHA-mH5-HA-VN and pHA-mH5-HA-IN.* For cloning the HA gene of A/Vietnam/1203/2004 (Accession number AY818135) the plasmid pDD4mH55TNT-VN-HA [15] was used. For cloning the HA gene of A/Indonesia/5/07 (HA-IN, Los Alamos data base ISDN125873) the pPCR-Script-HA-IN [15] was used. The HA-IN gene was placed downstream of an early/late promoter in pDD4-mH5-5TNT resulting in pDD4-mH5-HA-IN. The pDD4-mH5-5TNT is a recombination plasmid directing the D4/D5 intergenic region of vaccinia virus. It codes for the essential D4R gene in reverse orientation. Foreign genes cloned into the multiple cloning site of pDD4-mH5-5TNT will be expressed under the synthetic early/late promoter mH5 [17]. The vectors contain also the vaccinia virus stop-signal TTTTTNT (5TNT) [18]. The HA-expression cassettes were amplified by PCR using the primer o.MVA-1 (5′-TTT CAC TAA AGC TGC GGC CG-3′) and o.MVA-2 (5′-CCG AAA CGC CGT TAA CAA AA-3′) from the templates pDD4mH55TNT-VN-HA or pDD4-mH5-HA-IN and ligated in pHA-vA by using Eco105I (Fermentas). The final constructs were characterized by restriction enzyme analysis and verified by sequencing. *pHA-mH5-HA-AN, pHA-mH5-HA-CE and pHA-mH5-HA-TT.* The HA-sequences of the A/Anhui/1/2005(H5N1) strain (accession number DQ371928) and the A/chicken/Egypt/3/2006(H5N1) strain (Accession number EU146866) were chemically synthesized (Geneart, Inc., Regensburg, Germany). Each fragment was cloned into the plasmid pER-mH5-PL [19] using NcoI and XmaI restriction sites, resulting in pER-mH5-HA-AN or pER-mH5-HA-CE. The plasmid pER-mH5-PL was obtained by insertion of the vaccinia virus promoter mH5, a vaccinia virus stop signal (TTTTTNT) and a multiple cloning site (StuI, NcoI, PvuII, SpeI, HindIII, SacI, XmaI, SalI, NotI) into plasmid pER that directs the gene cassette into the D4/D5 intergenic region [19]. The RNA of Influenza A/turkey/Turkey/1/2005 (H5N1, Accession number EF619980) was isolated and used for cDNA synthesis with the primer o.FluRT-2 (5′-AGC AAA AGC AGG GGT ATA ATC TGT C-3′). PCR was performed by using the primers o.HAv-10 (5′-ACC ATG GAG AAA ATA GTG CTT C-3′) and o.HAu-2 (5′-GTC GAC TTA AAT GCA AAT TCT GCA TTG TAA C-3′) from this cDNA. The PCR-fragment was cloned in pDD4-mH5-5TNT by using NcoI and SalI restriction sites, resulting in pDD4-mH5-HA-TT. The HA-expression cassettes were amplified by PCR using the primers o.pER-mH5 5′ XhoI (5′-GAC TCT CG AGG CAG CTA GCA AAA ATT GAA A 3′-) and o.pER-3′ BamHI (5′-GTA TGG ATC CAA ATT TCA CTA AAG CTG CGG-3′) from pER-mH5-HA-AN, pER-mH5-HA-CE and pDD4-mH5-HA-TT, respectively. The PCR-fragments were cut using restriction enzymes XhoI and BamHI and ligated in pHA-vA by using the same restriction sites. The final constructs were characterized by restriction analyses and verified by sequencing of the inserted DNA.

### Construction of recombinant MVA

*MVA-mH5-HA-VN, MVA-HA-IN, MVA-HA-AN, MVA-HA-CE, MVA-HA-TT.* Twenty micrograms of pHA-mH5-HA-VN, pHA-mH5-HA-IN, pHA-mH5-HA-AN, pHA-mH5-HA-CE, or pHA-mH5-HA-TT plasmid DNA were transfected into MVA infected primary chicken cells (CEC) by calcium phosphate precipitation and further processed as described previously [20]. The purified recombinant virus isolates were expanded for large-scale preparations in CEC.

### MVA titration by plaque-assay

The virus stocks were characterized by defining the virus amounts in 1 ml stock solution. Plaque titrations of recombinant MVA was performed in DF-1 cells. Serial ten-fold dilutions were made and used for infection DF-1 cells cultivated in six well plates. Two wells per plate were infected with the same virus dilution. Three days after infection, plaques were stained using 0.1% crystal violet dissolved in a 20% ethanol solution. The titer was calculated by number of plaques multiplied by dilution.

### SDS-Page and Western Blot

Expression of the HA proteins by MVA was detected by Western blotting. DF-1 cells were infected with a MOI of 0.1 for 48 hours. Infected cells were harvested by scraping and mixed with 2x Laemmli buffer (Fermentas, Inc.). To separate the proteins the cell lysates were loaded on 12% Tris-gels (BioRad, Inc) together with molecular weight standards (PageRuler™ Prestained Protein Ladder; Fermentas). After transfer to a nitrocellulose membrane (Invitrogen, Inc), total protein was stained with Ponceau-S-Solution (Sigma, Inc). The HA proteins were detected with a 1∶1000 solution of a sheep antiserum against the A/Vietnam/1194/04 (H5N1) hemagglutinin (National Institute for Biological Standards and Control, NIBSC 04/214). A 1∶2000 dilution of a donkey-anti-sheep alkaline phosphatase-conjugated IgG (Sigma Inc) was used as a secondary antibody.

### Double immunostain assay

DF-1 cells were infected with 10, 100 and 1000 pfu of recombinant MVA. The infection was overlaid with 3% carboxymethyl cellulose (CMC, Sigma, Inc). After 4 days infection, the cells were fixed with methanol/acetone solution. The HA-Protein was stained using a 1∶1000 solution of a sheep antiserum against the A/Vietnam/1194/04 (H5N1) hemagglutinin (National Institute for Biological Standards and Control, NIBSC 04/214) as first antibody and an anti sheep IgG, HRP conjugated antibody (Sigma, Inc.) as second one. The HA-Protein staining was done with a DAB-staining kit (Vector Lab) containing nickel resulting in black-grey colored plaques. Afterwards the vaccinia virus proteins in recombinant MVA infected cells were stained. The vaccinia virus proteins were detected by a rabbit anti vaccinia virus sera (produced in house, Baxter) as first antibody and with a peroxidase-conjugated goat anti rabbit antibody (Sigma, Inc) as second antibody. The staining was performed with a DAB staining solution kit (Vector Lab) without nickel. This staining resulted in brown staining distinguishable from the black-grey stained plaques.

### Particle Infectivity (PI) Ratio

The particle infectivity (PI) ratio, as a parameter for quality of a virus stock, was determined as described elsewhere [21]. Briefly, real-time PCR was used to determine the amount of MVA gene copies (genomic equivalents) and set in relation with the plaque forming units (pfu) of the stock.

### Protection/challenge experiments

Groups of 6 female Balb/c mice (6 to 7 weeks old, Charles River) were immunized intramuscularly once with 10^6^ pfu of recombinant MVA virus. Control groups were immunized with 10^6^ pfu wild-type (wt) MVA virus or with buffer. Blood samples were taken for IgG ELISA and HI titer determinations on days 20 and 41. Mice were challenged intranasally with 1×10^5^ TCID50/ml of the respective influenza viruses (A/Vietnam/1203/2004, A/Indonesia/5/05, A/Anhui/5/2005, or A/turkey/Turkey/1/05) on day 42 and monitored over 14 days. Symptoms including ruffled fur (score of 1), curved posture (score of 2), apathy (score of 3), and death (score of 4) were recorded. As outlined in ref. [15], the virus dose that kills 50% of the mice (LD_50_) is 24 TCID50 for the A/Vietnam/1203/2004, 18 TCID50 for A/Indonesia/5/07, 9 TCID_50_ for A/Anhui/5/2005 and 11 TCID_50_ for the A/turkey/Turkey/1/05 strain.

### Immunization experiments for cellular immunity

Groups of 5 female Balb/c mice (6 to 7 weeks old, Charles River) were immunized intramuscularly twice (days 0, 21) with 10^6^ pfu of recombinant MVA virus. Control groups were immunized with 10^6^ pfu wild-type (wt) MVA virus or with buffer. Spleens were obtained for IFN-γ analyses on day 28 post-immunization after euthanizing the mice.

### ELISA (enzyme linked immunosorbant assay)

The ELISA assay was done as described elsewhere [3]. Briefly, the ELISA titer was measured against recombinant H5 (rHA-H5 A/Vietnam/1203/2004, Protein Sciences Corporation). All sera were serial diluted in four-fold steps starting with 1∶100 dilution (1∶100 to 1∶409,600). After incubation with the HRP-labeled secondary anti-mouse IgG antibody (Sigma, Inc.) and color development using OPD/H_2_O_2_ plates were read at 490/620 nm. The antibody titer of the serum samples determined as reciprocal of the dilution step, which was equal or greater than the cut-off value. The cut-off value was four times the mean absorbance value of a negative control serum at a 1∶100 dilution.

### Micro neutralization (µNT) assay

The µNT assay was performed as described elsewhere [3]. Briefly, sera were serially diluted in two-fold steps. The diluted sera were mixed with the respective virus strains (A/Vietnam/1203/2004, A/Indonesia/5/05, A/Anhui/5/2005, or A/turkey/Turkey/1/05) at a concentration of 100 TCID50 per well, and transferred to a monolayer of Vero cells [22]. Five days post incubation, Vero cells were inspected for cytopathic effects. The neutralizing antibody titer was defined as the reciprocal serum dilution at which virus growth was 50% inhibited and was calculated by the method of Reed and Muench [23].

### T-cell IFN-γ analysis

Flow cytometric intracellular IFN-γ staining was used to determine frequencies of hemagglutinin-specific CD4 and CD8 T-cells in Balb/c mice 7 days after the second immunization as described previously [15]. Pooled splenocytes from 5 animals were analyzed in two independent immunization experiments. In each case, single cell suspensions of splenocytes were stimulated overnight in the presence of 10 µg/ml brefeldin A (Sigma) with 3 µg hemagglutinin per ml of inactivated whole virus antigens (H5N1/Vietnam/1203/2004, H5N1/Indonesia/5/05, H1N1/California/07/2009), or with two peptides from hemagglutinin (HA140-154, HA189-197) at 2 µg/ml per peptide. The 9-mer peptide HA189-197, IYSTVASSL, contains the immune dominant, H2-Kd restricted CD8 T-cell epitope that is highly conserved within the H1 or H5 hemagglutinins and is located in the HA2 subunit [24]. The 15-mer HA140-154 peptide, KSSFFRNVVWLIKKN, represents the dominant H2-d restricted CD4 T-cell epitope within the H5 hemagglutinin of the H5N1/Vietnam/1203/2004 strain. Cells were then incubated with LIVE/ Dead Violet Kit (VIVID, Molecular Probes) subsequently stained with rat anti-mouse CD4-APC and CD8-APC H7 antibodies and, after permeabilization with 0.08% saponin (Sigma), with rat anti-mouse IFN-γ FITC and CD3-PerCP antibodies (all BD Biosciences). At least 100 000 viable cells were acquired on a FACSCanto-2 (BD Biosciences), and analyzed using FlowJo software (Tree Star, Inc.). Percentages of IFN-γ producing T-cells were calculated after gating on VIVID negative, CD3 positive, CD4 or CD8 positive lymphocytes.

### T-cell epitope screen

One hundred eleven fifteen-mer peptides covering the whole H5/Vietnam hemagglutinin with an overlap of 5 amino acids were synthesized using tri-fluoroacetate as counterion (JPT Peptide Technologies GmbH, Germany). Purity of the peptides was >90% in all cases, and identity of the peptides was verified by mass spectrometry. Splenocytes obtained from Balb/C mice after 2 immunizations with vaccinia virus expressing H5 hemagglutinin of H5N1/Vietnam/1203/2004 were used to identify those peptides which could induce specific IFN-γ CD4 or CD8 T-cell responses upon restimulation by FACS analysis as described above.

### Statistical analysis

For statistical analysis of survival differences between animal groups, the Kaplan Meyer log rank test of GraphPad Prism software (San Diego, CA) was used. Data of viral micro NT, ELISA IgG titers and clinical score were analyzed using the one-way ANOVA and Tukeýs Multiple Comparison Test. All differences were considered significant at P<0.05.

## Results

### Construction and characterization of MVA viruses expressing different H5 hemagglutinins

According to the standard nomenclature system based upon the evolution of the H5 hemagglutinin genes, the H5N1 influenza viruses are grouped in different clades [25]. Within one clade the nucleotides must not differ more than 1.5%. In this study, the HA genes of five different clades with 95 to 99% identity on protein level were used to analyze cross protection in a mouse model. The influenza strains used and the amino acid identities are depicted in **Table 1**. The CE3/06 and TT01/05 hemagglutinins, for instance, differ only by one amino acid, whereas the IN5/05 and TT01/05 strains share only 95% identity.

**Table 1:** Amino acid sequence identities of influenza HA proteins of strains used in this study.

clade	Influenza strain	Identity between different strains in %				
		VN/1203	IN5/05	CE3/06	TT01/05	AH1/05
1	VN/1203(1)	100	96	96	96	96
2.1.3	IN5/05(2)	96	100	95	95	96
2.2.	TT01/05(3)	96	95	99	100	96
2.2.	CE3/06(4)	96	95	100	99	96
2.3.4	AH1/05(5)	96	96	96	96	100

(1) VN/1203, A/Vietnam/1203/2004(H5N1);

(2) IN5/05, A/Indonesia/5/05(H5N1);

(3) TT1/05, A/turkey/Turkey/1/2005(H5N1);

(4) CE3/06, A/Chicken/Egypt/3/2006(H5N1);

(5) AH1/05, A/Anhui/1/2005(H5N1).

For construction of the recombinant viruses, the HA genes were each inserted into the genomic 165R locus of MVA [26] by in-vivo recombination techniques (see methods) resulting in five candidate vaccines. The different HA genes were placed downstream of a strong vaccinia early/late promoter [17] and were optimized for expression by vaccinia virus removing internal RNA transcription stop signals [18]. Viruses were grown in primary chicken cells and purified by centrifugation through a sucrose cushion. All preparations had similar titers in the range of 5–8×10^9^ pfu/ml. Furthermore, the particle-to-infectivity ratio, determined by measuring the relation between genomic equivalents by real-time PCR and live virus by plaque assay, was similar among the recombinant viruses indicating comparable infectivity of the virus preparations (**Table 2**).

**Table 2:** Recombinant MVA viruses used for immunizations.

recombinant virus	HA gene of the strain (accession number)	recombination plasmid(1)	Titer(2) (pfu/ml)	PI-ratio(3)	HA exp. (%)(4)
MVA-HA-VN	A/Vietnam/1203/2004 (AY818135)	pHA-mH5-HA-VN	8.4×10^9^	58	100
MVA-HA-IN	A/Indonesia/5/05 (ISDN125873)	pHA-mH5-HA-IN	6×10^9^	45	100
MVA-HA-TT	A/turkey/Turkey/1/05 (EF619980)	pHA-mH5-HA-TT	5.1×10^9^	54	100
MVA-HA-CE	A/Chicken/Egypt/3/2006 (EU146866)	pHA-mH5-HA-CE	5.4×10^9^	34	100
MVA-HA-AN	A/Anhui/1/2005 (DQ371928)	pHA-mH5-HA-AN	5.8×10^9^	20	100

(1) All HA genes are controlled by the vaccinia virus mH5 promoter;

(2) titers of sucrose-purified virus preparations from CEC cells (see methods);

(3) particle infectivity ratio, ratio of genomic equivalents determined by qPCR to infectious virus particles (pfu);

(4) HA expression as determined by immunostaining (see methods).

To test for purity of the recombinant viruses, a double immunostain assay was performed. As described in methods, plaques were first stained against the influenza HA proteins (resulting in brown plaques) and afterwards against vaccinia virus proteins (resulting in black plaques in the wild-type viruses or nonexpressing mutants only) allowing us to distinguish HA-expressing viruses from non-expressing contaminants. As a control a mixture of recombinant and wild-type viruses was used. All recombinants were positive for the influenza HA and non-HA-expressing viruses were not detected (not shown). From this analysis it was concluded that all of the recombinant MVAs express the HA protein (**Table 2**). The genomic structure of the recombinant MVA viruses was confirmed by PCR analysis. A primer set flanking the insertion site of the foreign genes was used to show that the recombinant viruses had the expected enlarged fragment and were free of wild-type virus. An additional primer set with one specific primer binding within the foreign gene and one binding at the insertion site was used to identify the HA gene in the insertion locus (not shown).

### Expression of the hemagglutinins in chicken cells

**Figure 1:**
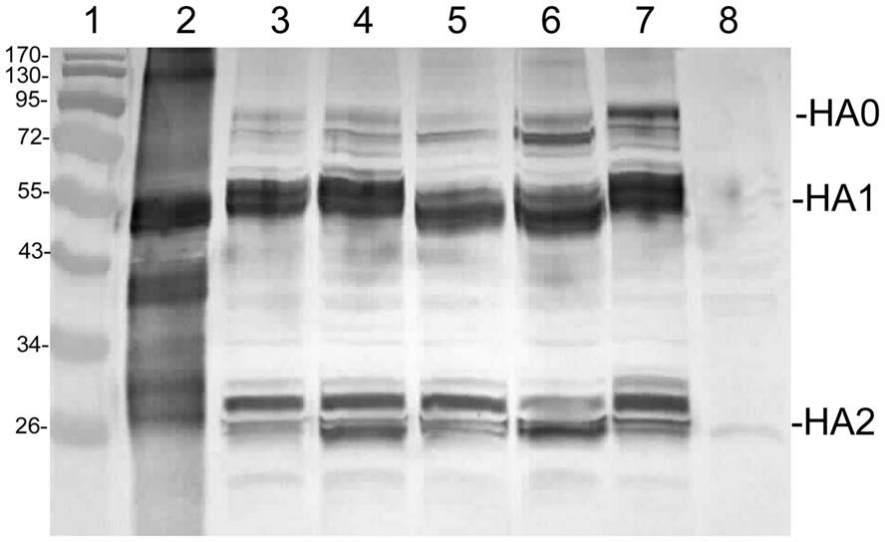
Western blot of chicken cell lysates tested for influenza virus HA expression. Lane 1, protein ladder, size in kDa; lane 2, positive control, 0.5 µg of formalin inactivated purified influenza virus A/Vietnam/1203/2004 H5N1. Lane 3, MVA-HA-VN. Lane 4, MVA-HA-IN. Lane 5, MVA-HA-TT. Lane 6, MVA-HA-CE. Lane 7, MVA-HA-AN. Lane 8, negative control, empty vector MVA wt. For abbreviations see Table 2. All recombinant MVAs (lanes 3–7) express the HA0 (band around 80 kDa), the HA1 (band around 55 kDa, and the HA2 (band around 25 kDa). The specific HA bands co-migrate with the ones of the positive control (lane 2) and are absent in the negative control (lane 8).

The correct expression of the influenza HA by the recombinant MVAs was analyzed by Western blotting. For this purpose the permanent chicken cell line DF-1 [27] was used. The cells were infected with a MOI of 0.1 and cell lysates were prepared 72 hrs post infection. The recombinant MVAs that express the different hemagglutinins were analyzed in a Western blot using an anti-influenza A/Vietnam/1194/04 (H5N1) polyclonal serum for detection (**Fig. 1**). Ponceau-S staining was used to assure that the total protein content in all lanes was equivalent after the transfer. The Western blot demonstrates that all recombinants express the influenza hemagglutinin, and that efficient processing of the protein takes place, resulting in similar bands (HA0, HA1, and HA2) as seen with the influenza control (compare lane 2 and **lanes 3–7**). The large band at 80 kDa represents residual uncleaved HA0. The lower bands around 55 and 26 kDa represent the HA1 and HA2 subunits. Due to the presence of a polybasic cleavage site in the H5 hemagglutinins, efficient cleavage is achieved also in non-respiratory epithelial cells, like the DF-1 cell line. The double band (in the 26 kDa size range) is presumably due to glycosylation variants of the HA2 subunit in the total cell lysates.

### Cross protection of mice from lethal challenge

**Figure 2:**
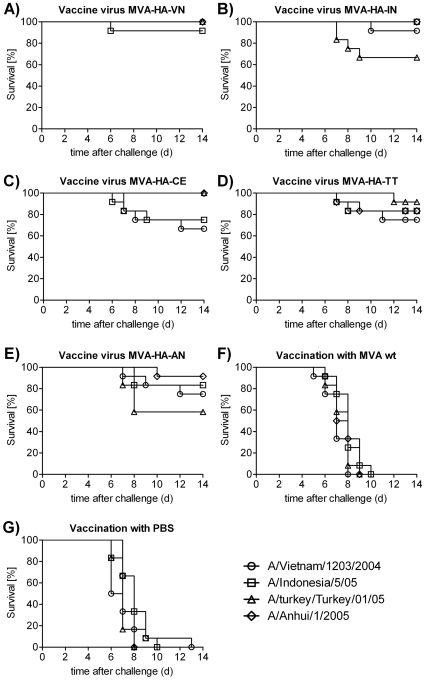
Survival after vaccination with recombinant MVAs and challenge with different H5N1 strains. For single dose vaccinations recombinant MVA vaccines expressing the HA of VN/1203 (A), of IN5/05 (B), of CE3/06 (C), of TT01/05 (D) and AH1/2005 (E) were used. As controls, mice were vaccinated with wt MVA (F) or were treated with PBS (G). After challenge with wild-type H5N1 strains of the different clades, mice were monitored for 14 days. The data represent two separate experiments with six animals per group.

A single dose immunization scheme was used to detect differences in protection among the HA proteins of the different clades. In pre-experiments it was found that double dose immunization results in robust protection, which precludes the detection of subtle differences in cross protection. To assess cross protection with the five MVA-based experimental vaccines, Balb/c mice were immunized once i.m. with a dose of 1×10^6^ pfu/animal and challenged intranasally 42 days later with the homologous or heterologous influenza wild-type strains. Two separate cross protection experiments were performed. In the pre-challenge sera, total anti-H5 IgG antibodies were determined by ELISA (see materials and methods). Neutralizing antibodies were assayed separately against each of the challenge strains (with the exception of the CE/06 strain, which was not available). The results, are presented in **Table 3**. Since the lethal dose 50 (LD50) of all H5N1 wild-type strains in Balb/c mice was similar (range 9–24 TCID_50_ per animal; see methods) a uniform challenge dose of 1×10^5^ pfu/animal was chosen for all strains. Survival and clinical scores were monitored over a period of 14 days and are depicted in **Figs. 2** **and** **3**).

**Figure 3:**
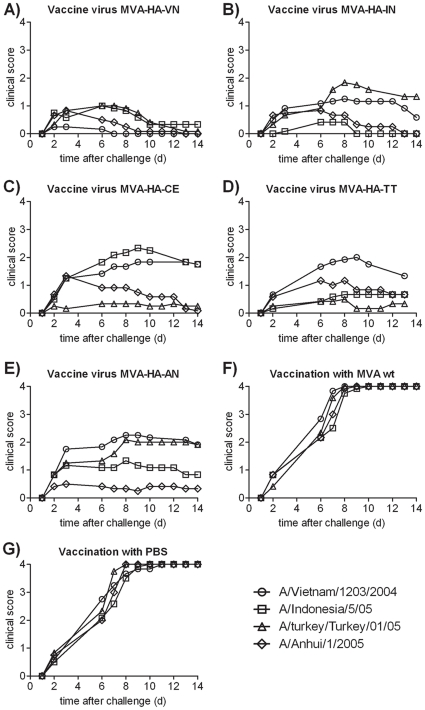
Clinical disease scoring after vaccination with recombinant MVAs and challenge with different H5N1 strains. MVA vaccines expressing the HA of VN/1203 (A), of IN5/05 (B), of CE3/06 (C), of TT01/05 (D) and AH1/2005 (E) were used for vaccination. Controls included wt MVA (F) or PBS (G). After challenge with wild-type H5N1 strains of the different clades, mice were clinically monitored for 14 days. The parameters used to evaluate the clinical score were ruffled fur (1 point), arched posture (2 points), apathy (3 points) and death (4 points). Each data point represents the arithmetic mean of two separate experiments with six animals per group.

**Table 3:** Protection of mice from death, clinical score and serology results after single dose vaccination with MVA H5 recombinants.

	Vaccine virus(1)	Challenge virus(2)	Survival n/nt(3) (%)	clinical score (4) (day 8/d14)	IgG [GMT]	µNT [GMT]
A	MVA-HA-VN(5)	VN/1203	12/12 (100)	0/0	4525	62
		IN5/05	11/12 (92)	0.84/0.34		17
		TT01/05	12/12 (100)	0.75/0.09		28
		AH1/2005	12/12 (100)	0.25/0		<14
B	MVA-HA-IN	VN/1203	11/12 (92)	1.25/0.59	1345	<7
		IN5/05	12/12 (100)	0.42/0		80
		TT01/05	8/12 (67)	1.84/1.33		28
		AH1/2005	12/12 (100)	0.67/0		13
C	MVA-HA-TT	VN/1203	9/12 (75)	1.92/1.34	1345	<7
		IN5/05	10/12 (83)	0.67/0.67		13
		TT01/05	11/12 (92)	0.5/0.34		67
		AH1/2005	10/12 (83)	1.17/0.67		<7
D	MVA-HA-CE(5)	VN/1203	8/12 (67)	1.67/1.75	3805	<7
		IN5/05	9/12 (75)	2.17/1.75		<14
		TT01/05	12/12 (100)	0.34/0.25		37
		AH1/2005	12/12 (100)	0.92/0.09		<14
E	MVA-HA-AN	VN/1203	9/12 (75)	2.25/1.92	4525	<7
		IN5/05	10/12 (83)	1.34/0.84		17
		TT01/05	8/12 (67)	2.09/1.92		12
		AH1/2005	11/12 (92)	0.36/0.33		22
F	MVA	VN/1203	0/12 (0)	4/4	<100	<7
		IN5/05	0/12 (0)	3.75/4		<7
		TT01/05	0/12 (0)	4/4		<7
		AH1/2005	0/12 (0)	3.92/4		<7
G	PBS	VN/1203	0/12 (0)	3.67/4	<100	<7
		IN5/05	0/12 (0)	3.5/4		<7
		TT01/05	0/12 (0)	4/4		<7
		AH1/2005	0/12 (0)	4/4		<7

(1) Mice were vaccinated once with 10^6^ pfu of recombinant MVA or wt MVA; for abbreviations see Tables 2 and 3;

(2) challenge dose, 1×10^5^ TCID_50_; for abbreviations see Table 1;

(3) n/nt, number of survivors/total animals of two separate experiments with 6 animals per group;

(4) clinical score at day 8 (peak)/day 14 (the end of experiment) after challenge. Each number represents the arithmetic mean from 12 animals;

(5) final serum dilutions in µNT assay were 1∶20 (dl 14), in all other sera 1∶10 (detection limit <7).

The *MVA-HA-VN* candidate vaccine induced excellent protection from death against all clades (**Table 3A** **,** **Fig. 2A**). After challenge with the homologous VN/1203 strain all animal survived and very few clinical symptoms were recorded (**Fig. 3A**). With the more distantly related Turkey/turkey and Anhui strains, full survival with some minor clinical symptoms was seen. Against challenge with the Indonesia strain 92% survival was achieved. The IgG titer induced by immunization (geometric mean titer, GMT, from two determinations) was high. Neutralizing antibodies, using the different H5N1 viruses in the NT assay, were measurable, with the exception of the Anhui strain, which represents the most distant clade of the tested viruses.

The results obtained after immunization with the *MVA-HA-IN* vaccine are shown in **Table 3B** and **Figs. 2B** **and** **3B**. This vaccine induced full protection from death against homologous challenge and against the heterologous Anhui strain with some minor clinical symptoms. Survival was 92% after challenge with the VN/1203 strain, however, only 67% of the animals survived challenge with the more distant (clade 2.2) TT01/05 strain. Neutralizing antibodies were higher against the homologous virus and lower against the strains of the other clades.

Animals that had received the vaccine based on the TT01/05 strain, *MVA-HA-TT* (**Table 3C** and **Figs. 2D** **and** **3D**), showed almost full survival (92%) with low clinical scores, while survival after challenge with the most distant VN/1203 virus was only 75%, and stronger clinical symptoms were seen. Protection from death against the more related clades IN5/05 and AH1/2005 was 83%. Neutralizing antibodies were elevated against the homologous TT01/05 wt virus used in the NT assay, and lower against the IN5/05 virus and below detection in the distantly related viruses.

The clade 2.2 chicken Egypt recombinant virus, *MVA-HA-CE*, conferred full protection from death against challenge with the most closely related clades, TT and Anhui (**Table 3D** **and** **Figs. 2C** **and** **3C**). Although all mice developed clinical symptoms within three days post challenge, this was not associated with substantial lethality. The vaccine was partially protective against the more distant clades (IN and VN) with maximal survival rates of 75% and higher clinical scores. Since no wt CE virus was available, homologous challenge could not be carried out. Neutralizing antibodies were detectable against the TT01/05 strain and were below the detection limit with the other strains.

The protection profile of the clade 2.3.4 Anhui based recombinant, *MVA-HA-AN*, was similar to the TT recombinant, with 92% survival after homologous challenge and maximal 83% cross protection from death against the other clades (**Table 3E** and **Figs. 2E** **and** **3E**). Although IgG ELISA antibodies were high, no neutralizing antibodies were detectable. Also, neutralization of the homologous virus was moderate, consistent with the protection results. All mice in the control groups immunised with MVA wt (**Tab. 3F** **,** **Figs. 2F** **and** **3F**) or with PBS (**Tab. 3G** **,** **Figs. 2G** **and** **3G**) were not protected. From day two on infected mice developed severe clinical symptoms and died within 6 and 10 days. Consistent with this finding, the ELISA and NT titers were below the detection limits.

### T-cell responses induced by the MVA-based vaccines

Vaccine-specific T-cell responses were determined by intracellular cytokine staining and FACS analyses. First, a T-cell epitope screen with 15-mer peptides covering the whole H5/Vietnam hemagglutinin with an overlap of 5 amino acids was used to identify those peptides within HA which elicit a CD4 or CD8 T-cell response after vaccination with vaccinia-based live vaccines. As expected, a single peptide (HA185-199 QILSIYSTVASSLAL) completely represented the CD8 response induced by a peptide pool containing all 111 peptides (data not shown), as this peptide contains the IYSTVASSL motif, the immune dominant, H2-Kd restricted CD8 T-cell epitope that is highly conserved within the H1 or H5 hemagglutinins and which is located in the HA2 subunit [24]. Interestingly, however, it was found that CD4 T-cells directed against the HA of the VN/1203 strain also specifically recognized a single peptide, identified as the 15-mer HA140-154 peptide KSSFFRNVVWLIKKN. This peptide is well conserved among the H5 variants but not in H1 (**Table 4**) and was unable to restimulate HA-1/CA/07 specific CD4 T-cells (data not shown).

**Table 4:** Amino acid sequences of the CD4 T cell-specific 15mer peptide HA140-154 (located in the HA1 subunit) in the different hemagglutinins.

strain	Sequence
VN/1203	K S S F F R N V V W L I K K N
IN5/05	S P - - - - - - - - - - - - -
TT01/05	R - - - - - - - - - - - - - D
CE3/06	R - - - - - - - - - - - - - D
AH1/05	T P - - - - - - - - - - - - -
CA/07	A K - - Y K - L I - - v - - G

To compare the efficiencies of the various MVA based vaccines to induce influenza-specific T-cell responses, groups of Balb/c mice were immunized in a two-dose regimen on days 0 and 21 with 10^6^ pfu of the recombinant MVA virus vaccines or the wild-type MVA control, and spleens were collected on day 7 after the first or second second immunization. Whole viral antigens from H5N1 of different clades, i.e. VN/1203 and IN5/05, and from H1N1/California (CA/07) were used for re-stimulation of the splenocytes to investigate the induction of cross-reactive cellular immune responses. In addition, the known cross-reactive CD8 T-cell peptide, which is not only conserved within the H5 strains, but also among the H1 hemagglutinins, and the newly defined CD4 peptide from H5/Vietnam were used. After a single immunization, HA specific CD8 T-cell responses were readily detectable and comparable for all H5-expressing viral vaccines, ranging between 0.27% and 0.39%. In contrast, levels of HA-specific CD4 T-cells were generally very low being at or just above the detection limit of the assay (0.05%). Since the inherent variability of the test results at low response levels did not allow for accurate comparison of the various vaccines, only the higher responses obtained after a booster immunization were compared.

**Figure 4:**
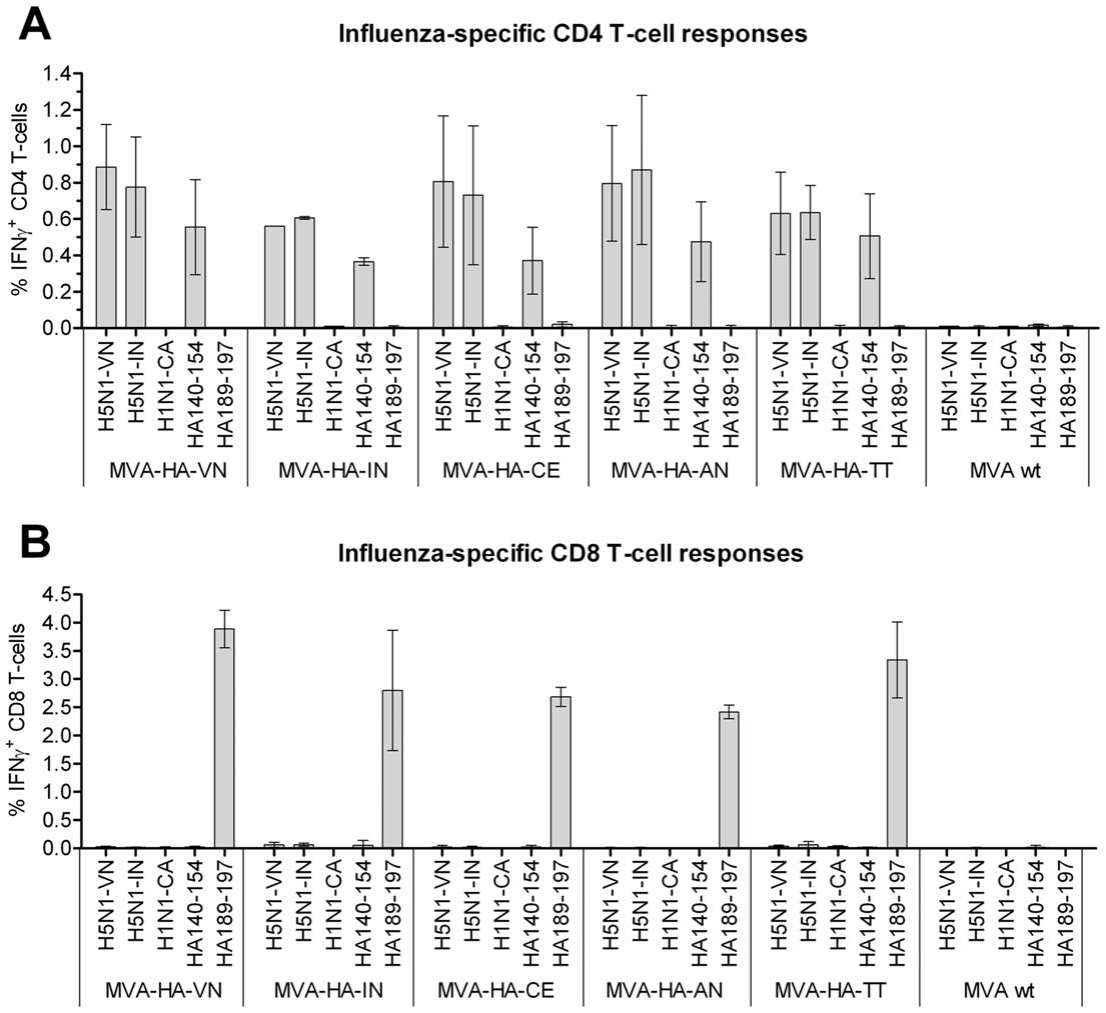
Induction of influenza-specific T cells by the hemagglutinin expressing live vaccines. Frequencies of antigen specific IFN-γ+ CD4 T cells (A) or CD8 T-cells (B) after immunizing twice with various hemagglutinin MVA-H5 vaccines or with the MVA wild-type control, and stimulation with protein antigens and peptides (shown on x-axis). Splenocytes were stimulated with buffer or with formalin-inactivated whole viral antigens of H5N1 Vietnam (H5N1-VN), H5N1 Indonesia (H5N1-IN) or H1N1/California (H1N1-CA), CD4 T cell-reactive peptide HA140-154 and the CD8 T cell-reactive peptide HA189-197. Background medium responses were subtracted, and the means +/− standard deviation of two separate experiments is shown.

As illustrated in **Fig. 4A**, both H5N1 whole viral antigens, but not the H1N1/California antigen, induced strong IFN-γ secretion in CD4 T-cells of mice immunized with the experimental recombinant vaccines. On average, between 0.6 and 0.9% of the CD4 T-cells responded to the two H5N1 whole virus antigens in two independent experiments. No significant difference was observed in the reactions against the Vietnam and Indonesia antigens. Furthermore, CD4 induction was similarly strong after stimulation with peptide HA140-154 in all the MVA vaccine immunization groups. The frequencies detected were only slightly lower than those found when using the whole viral antigens, indicating that this peptide represents the most prominent CD4 T cell epitope present within the HA molecule. The corresponding peptide sequence is substantially mutated in HA from H1/California (**Table 4**), and is not recognized by HA/Ca-specific CD4 T-cells induced after immunization with H1N1/California whole viral vaccine (data not shown). As expected, the CD8 T cell-specific 9-mer peptide HA189-197 did not stimulate any CD4 T-cell responses, nor did the MVA wild-type virus induce detectable frequencies of influenza-specific CD4 T-cells in the mice.

When the CD8 T-cell responses were analyzed, only the 9-mer peptide HA189-197 induced a strong IFN-γ response (**Fig. 4B**). Similarly, high frequencies of HA-specific CD8 T-cells were induced by all the vaccines, with responses ranging from 2.5 to 4% of total CD8 T-cells. Hence, there appeared to be little or no difference in the efficiency of the various vaccines to induce CTL responses. In contrast, as anticipated, no response was observed when the wild-type MVA vaccine was used for immunization.

### Mapping of the amino acid differences in the hemagglutinins in a three-dimensional model

**Figure 5:**
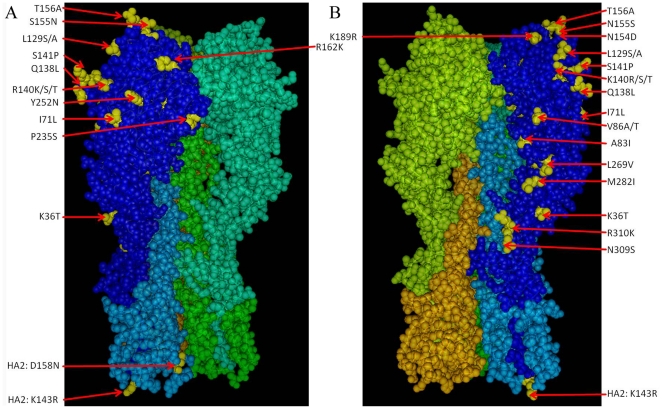
Three dimensional pictures of the HA trimers of the avian subtype 5 hemagglutinin. Amino acids differing from the HA of VN/1203 are marked yellow in one of the HA monomers. A and B represent two different views of the trimer. Amino acids exposed on the surface are marked in yellow and are printed in bold in Table 5.

An attempt was undertaken to map and interpret the amino acid differences in the hemagglutinins in a three-dimensional model of the subtype 5 HA trimer of the VN1194 strain [28]. The HA of VN/1194 differs from that of VN/1203 by only one amino acid (T36K) and is therefore an excellent representative of a clade 1 hemagglutinin. As the 3 D structure of the VN/1194 HA is present online (protein database, PDB ID 2IBX), interpretation was readily feasible. All amino acids exposed on the surface of the HA trimer were mapped in the 3D model, with the space filling mode of the amino acid molecules chosen to display the HA trimer (**Fig. 5**). All differences between the five HAs were highlighted in yellow in one of each of the HA1 and HA2 molecule (**Fig. 5A, B**). Differing amino acids exposed to the surface of the molecule are listed in bold in **Table 5**. Twenty one of the 36 amino acids that differ between the five different HAs are exposed to the surface. Most of these differences are located at or near the receptor binding site.

**Table 5:** Differences in the HA amino acid sequence compared to the VN/1203 master sequence.

	…	36^1^	…	…	71	…	…	83	…	…	86	…	…	94	…	…	124	…	…	129	…	…	138	…	140	141	…	…	154	155	156	…
VN^3^	-	**K** 2	-	-	**I**	-	-	**A**	-	-	**V**	-	-	D	-	-	S	-	-	**L**	-	-	**Q**	-	**K**	**S**	-	-	**N**	**S**	**T**	-
IN	-	**T**	-	-	**I**	-	-	**A**	-	-	**T**	-	-	S	-	-	D	-	-	**S**	-	-	**L**	-	**S**	**P**	-	-	**N**	**S**	**T**	-
CE	-	**T**	-	-	**L**	-	-	**I**	-	-	**A**	-	-	N	-	-	D	-	-	**S**	-	-	**Q**	-	**R**	**S**	-	-	**D**	**N**	**A**	-
TT	-	**T**	-	-	**L**	-	-	**I**	-	-	**A**	-	-	N	-	-	D	-	-	**A**	-	-	**Q**	-	**R**	**S**	-	-	**D**	**N**	**A**	-
AN	**-**	**T**	**-**	**-**	**I**	**-**	**-**	**A**	**-**	**-**	**A**	**-**	**-**	**N**	**-**	**-**	**D**	**-**	**-**	**S**	**-**	**-**	**Q**	**-**	**T**	**P**	**-**	**-**	**N**	**N**	**T**	**-**

	…	162	…	…	174	…	…	181	…	…	189	…	…	200	…	…	212	…	…	227	…	…	235	…	…	252	…	…	263	…	265	…	…	269	…
VN	-	**R**	-	-	V	-	-	P	-	-	**K**	-	-	V	-	-	R	-	-	E	-	-	**P**	-	-	**Y**	-	-	T	-	M	-	-	**L**	-
IN	-	**K**	-	-	V	-	-	P	-	-	**R**	-	-	I	-	-	K	-	-	E	-	-	**P**	-	-	**Y**	-	-	A	-	M	-	-	**L**	-
CE	-	**R**	-	-	V	-	-	P	-	-	**R**	-	-	V	-	-	K	-	-	E	-	-	**S**	-	-	**N**	-	-	T	-	M	-	-	**L**	-
TT	-	**R**	-	-	V	-	-	P	-	-	**R**	-	-	V	-	-	K	-	-	E	-	-	**P**	-	-	**N**	-	-	T	-	M	-	-	**L**	-
AN	-	**R**	-	-	I	-	-	S	-	-	**K**	-	-	V	-	-	K	-	-	D	-	-	**P**	-	-	**Y**	-	-	A	-	V	-	-	**V**	-

	…	282	…	…	309	310	…	…	322	323	…	325^4^	…	…	143^5^	…	…	152	…	…	158	…	…	183	…	…	203	…	…	207	…
VN	-	**M**	-	-	**N**	**R**	-	-	Q	R	-	R	-	-	**K**	-	-	V	-	-	**D**	-	-	T	-	-	V	-	-	S	-
IN	-	**M**	-	-	**N**	**R**	-	-	Q	R	-	S	-	-	**K**	-	-	I	-	-	**N**	-	-	I	-	-	M	-	-	S	-
CE	-	**I**	-	-	**N**	**R**	-	-	Q	G	-	R	-	-	**R**	-	-	V	-	-	**D**	-	-	I	-	-	V	-	-	F	-
TT	-	**I**	-	-	**S**	**R**	-	-	Q	G	-	R	-	-	**R**	-	-	V	-	-	**D**	-	-	I	-	-	V	-	-	S	-
AN	-	**I**	-	-	**N**	**K**	-	-	L	R	-	R	-	-	**K**	-	-	V	-	-	**D**	-	-	I	-	-	V	-	-	S	-

(1) HA1 numbering starts with the first amino acid downstream of the signal peptide with D as the first residue (Aichi numbering);

(2) amino acids exposed on the surface are printed in bold (equivalent to yellow marked molecules in Fig. 5);

(3) VN, A/Vietnam/1203/2004; IN, A/Indonesia/5/05; AN, A/Anhui/1/2005; CE, A/Chicken/Egypt/3/2006; TT, A/turkey/Turkey/1/2005;

(4) first R residue of polybasic cleavage site;

(5) numbering of the HA2 starts downstream of the polybasic cleavage site with G as the first residue.

## Discussion

For a direct comparison of the cross protective capacity of HA antigens from different clades, the HA genes were engineered into otherwise identical recombinant MVA vectors that were then used for immunization experiments. This approach allows analysis of the humoral immune response to the HA antigens independently of the influenza virus background. The in-vivo expression of the influenza surface proteins by the non-pathogenic live MVA vector further allowed to investigate the induction of a specific cellular immune response by the H5 hemagglutinins.

For the comparison of the humoral immune responses against the hemagglutinins, a suboptimal immunization protocol was used. In a previously published MVA-based immunization approach, robust protection against homologous challenge with 10^3^ TCID_50_ of A/Vietnam/1194/04 was found in C75BL6 mice [12]. In our setting using Balb/c mice, a 100-fold higher challenge dose of 10^5^ TCID_50_ was applied to measure the different degrees of protection achieved with the selected HA constructs. The LD_50_ of all employed challenge strains of influenza was similar, so that the infection dose of 10^5^ TCID_50_ consistently resulted in 100% lethality among the control animals. Immunized groups were protected to various extents, depending on the vaccine and the challenge virus chosen. When immunized animals were challenged with influenza viruses homologous to the HA used for immunization, 92% to 100% survival was seen, with at least some of the animals per group displaying moderate disease symptoms. However, when animals were challenged with influenza virus from heterologous clades, survival rates were reduced. The best protection against heterologous clades was conferred by MVA-HA-VN, which expresses the HA of the clade 1 A/Vietnam/1203/2004 strain. Similarly high cross protection was seen with the clade 2.1.3 A/Indonesia/5/05 (IN5/05) expressing recombinant MVA-HA-IN. In most cases, the degree of protection was in agreement with distance in the phylogenetic tree. Interestingly, cross-protection was not mutual in all cases. While all animals that had been immunized with the clade 1 and 2.1. constructs, MVA-HA-VN and MVA-HA-IN, survived challenge with the clade 2.3.4 A/Anhui/1/2005(H5N1) virus with minor disease symptoms, only 67-83% of MVA-HA-AN immunized animals survived challenge with A/Vietnam/1203/2004(H5N1) or A/Indonesia/5/05(H5N1). Since the vectors were equally effective in expressing the influenza HA antigens in vivo, the differences in the cross-protective effect are to be found in the antigenicity of the specific HA protein, i.e. the presence of immunogenic epitopes, and in the relation among the different clades. To assess the importance of the humoral immune response in this live vector approach, neutralizing antibody responses were measured against most influenza viruses used in the challenge study. Because a suboptimal immunization scheme was chosen to be able to discriminate the protection results by the different immunization/challenge virus pairs, the serological titers achieved by immunization with the experimental live vaccines were also lower than in a robust protective, two dose immunization regimen. Because of the low levels of neutralizing antibodies no correlation between protection and antibody titer could be determined.

The cellular immunity induced by the different HA antigens was also assessed. When harvested splenocytes were stimulated with whole virus antigens from clade 1 VN/1203, and from clade 2.3.1 IN5/05, respectively, very similar frequencies of IFN-γ-secreting CD4 T cells were observed, whereas stimulation with a H1N1 whole virus antigen revealed no measurable cellular immune response. Although the CD4-driven cellular immune response plays an important role in the defense against influenza [29], it is concluded that it does not contribute to the clade-specific differences in protection. Measurement of T cell responses by intracellular cytokine assay confirmed that all experimental vaccines induced high frequencies of both CD4 T-cells, which were specific for an HA1 epitope located within peptide HA140-154, and CD8 T-cells, which were directed against the well-known immune-dominant peptide HA189-197 located in the HA2 subunit. Since these responses were directed against T-cell epitopes conserved within the H5 hemagglutinin proteins and since influenza-specific CD4- as well as CD8 T cells can contribute to protection against influenza infection [30], it can be speculated that the heterologous protection observed despite the presence of only low levels of neutralizing antibodies was, at least in part, due to the cross-reactive cell-mediated immunity. However, as the frequencies of cross-reactive T-cells were very similar in all groups immunized with the MVA-based vaccines, these cells probably do not account for the distinct cross-protective efficiencies of the various antigens.

Numerous studies indicate an encouraging extent of cross-neutralization between various H5N1 strains but also stress that protection against clade 2.3 viruses may be more difficult [31]. The genetic polymorphisms between different clades of H5N1 viruses from 2002 to 2007 were extensively analyzed in a further publication, indicating that among others, HA residues at positions 86, 124, 129, 189, 212 and 263 constitute important antigenic sites (defined in HI assays) and that manipulation of these sites can improve cross-clade antigenic reactivity among diverse H5N1 strains [32]. Although VN/1203 differs in most of these positions from the other isolates analyzed it has nevertheless induced the best cross-protection in our study. Interestingly, recombinant MVAs expressing the HAs of strains CE3/06 and TT01/05, which exhibit D154, N155 and A156 residues and therefore do not carry a glycosylation motif in this region [33], show a tendency to be less protective against strains that carry this motif (IN, VN and AH), and vice versa. Residues at position 129 [34], [35] and 94 [36] have been described to alter receptor binding specificities but seem to have no impact on cross-protection in our analysis. In addition, the amino acids of the 140 s loop (positions 136 – 141), which has been shown to be a target for neutralizing antibodies [37] and is rather different in the analyzed HA proteins, also do not influence cross-protection significantly. Less substitutions are found in the stem region where more conserved antibody binding sites [38], and conserved CD8 T cell epitopes [39], [40], are located (for instance the 9mer HA189-197 used in this study). Virus neutralization is not only effected by antibodies that bind to the receptor binding region, but also by antibodies binding in the conserved HA2 region, that, for instance, block the pH-dependent conformational change required for membrane fusion and infectivity [38]. There were four changes in the conserved HA2 region, two of them exposed to the surface according to the 3 D model. Interestingly, the most abundant type of mutation is a K to R (Lys to Arg) mutation and vice versa, which occurred six times at different locations in the HA molecules. This mutation, caused by a single nucleotide change, is a structural change that does not disrupt the overall protein structure and seems to be one of the driving forces in shaping the structure of the related H5 proteins. In general, the superiority in cross protection induced by A/Vietnam/1204/04 hemagglutinin over the other tested hemagglutinins in our analysis could not be pin pointed to specific amino acid residues, but appears to be reflected by the whole antigenic profile of the protein. Regarding the H5 clades that were examined in this study, an MVA vector-derived H5N1 vaccine based on the VN/1203 strain would be the vaccine of choice.

By the use of MVA-based experimental vaccines, the cross-protective properties of HA antigens from different clades could be analyzed. Differences between clades were primarily attributed to neutralizing antibodies rather than to the cellular immune responses, because the cell-mediated immune responses were strong, but almost uniform among the H5 antigens, independent of the degree of homology among the clades. This finding suggests that HA-expressing live viral vectors are capable of triggering a strong and very broad cellular immune response against influenza viruses of different clades. Therefore MVA-based recombinant pandemic vaccines might represent a useful vaccine approach against influenza viruses especially in a pre-pandemic situation where the eventual pandemic virus target strain is not known.
